# Lipid nanoparticles loading triptolide for transdermal delivery: mechanisms of penetration enhancement and transport properties

**DOI:** 10.1186/s12951-018-0389-3

**Published:** 2018-09-15

**Authors:** Yongwei Gu, Meng Yang, Xiaomeng Tang, Ting Wang, Dishun Yang, Guangxi Zhai, Jiyong Liu

**Affiliations:** 1 Department of Pharmacy, Fudan University Shanghai Cancer Center, Department of Oncology, Shanghai Medical College, Fudan University, Shanghai, 200032 China; 2 Department of Pharmacy, Changhai Hospital, Second Military Medical University, Shanghai, 200433 China; 3 College of Pharmacy, Shandong University of Traditional Chinese Medicine, Jinan, 250355 Shandong China; 4 Department of Pharmacy, Shanghai Ninth People Hosipital, Shanghai Jiao Tong University, Shanghai, 200011 China; 5 Department of Pharmaceutics, College of Pharmacy, Shandong University, Jinan, 250012 Shandong China

**Keywords:** Lipid nanoparticles, Transdermal drug delivery system, Triptolide, Mechanisms of penetration enhancement, Transport properties

## Abstract

**Background:**

In recent years, nanoparticles (NPs) including nanostructured lipid carries (NLC) and solid lipid nanoparticles (SLN) captured an increasing amount of attention in the field of transdermal drug delivery system. However, the mechanisms of penetration enhancement and transdermal transport properties of NPs are not fully understood. Therefore, this work applied different platforms to evaluate the interactions between skin and NPs loading triptolide (TPL, TPL-NLC and TPL-SLN). Besides, NPs labeled with fluorescence probe were tracked after administration to investigate the dynamic penetration process in skin and skin cells. In addition, ELISA assay was applied to verify the in vitro anti-inflammatory effect of TPL-NPs.

**Results:**

Compared with the control group, TPL-NPs could disorder skin structure, increase keratin enthalpy and reduce the SC infrared absorption peak area. Besides, the work found that NPs labeled with fluorescence probe accumulated in hair follicles and distributed throughout the skin after 1 h of administration and were taken into HaCaT cells cytoplasm by transcytosis. Additionally, TPL-NLC could effectively inhibit the expression of IL-4, IL-6, IL-8, IFN-γ, and MCP-1 in HaCaT cells, while TPL-SLN and TPL solution can only inhibit the expression of IL-6.

**Conclusions:**

TPL-NLC and TPL-SLN could penetrate into skin in a time-dependent manner and the penetration is done by changing the structure, thermodynamic properties and components of the SC. Furthermore, the significant anti-inflammatory effect of TPL-NPs indicated that nanoparticles containing NLC and SLN could serve as safe prospective agents for transdermal drug delivery system.

## Background

Triptolide (TPL), a diterpene lactone epoxide compound extracted from the Traditional Chinese Medicine *Tripterygium wilfordii Hook F* (TWHF), is widely used to treat inflammation, autoimmune diseases, malignancy and depression [1–4]. Generally, TPL is recommended for oral administration. However, TPL rapidly reaches C_max_ (10 min), distributes in the other organs and excreted (t_1/2_, 38 min) via biliary, urinary and fecal routes after oral administration [5]. The toxic and side effects on liver and spleen were reported frequently [6, 7]. Thus, TPL formulation with better patient compliance and controlled release required more studies in novel drug delivery routes, including transdermal delivery.

Compared with the oral route, transdermal delivery are characteristic of providing long-time drug release and improving patient compliance. However, the primary permeability barrier for transdermal drug preparation is from the stratum corneum (SC). Through in-depth study of transdermal delivery, it developed from the first generation transdermal patches with little or no enhancement; through the second generation chemical enhancers, iontophoresis and liposomes for delivering small molecules; to the third generation physical enhancers combined with ultrasound, thermal ablation and microneedles for macromolecule [8]. In the three-generation transdermal preparations, the first generation is based on passive diffusion with poor drug penetration; the second generation of physical penetration-enhancing techniques including iontophoresis, electroporation, laser ablation, microneedle can be effective promote small molecule permeability, but has the disadvantage of high cost [9]; the third generation combined promotion technology can effectively enhance the macromolecular penetration, but there are shortcomings such as high requirements on equipment and patients can’t achieve self-administration.

Liposomal formulations as the second generation transdermal delivery are approved by FDA [10]. However, there are some disadvantages for liposomes, including lower drug loading, residue of organic solvent, not suitable for encapsulating biological fluids and aqueous solutions [11, 12]. To overcome these limitations, researches towards novel and advanced lipid nanoparticles (NPs), as those known as solid lipid nanoparticles (SLN) and nanostructured lipid carriers (NLC). Besides, the NPs could be used for improvement the solubility of poorly soluble drugs [13, 14]. TPL, the model drug of the research, is also water insoluble drug (5.95 ± 0.48 μg/mL) according to our previous study. So, loading TPL in NPs might be a strategy for improving its solubility.

NPs, first proposed in 1900s, can be categorized as SLN and NLC by the phase state of the lipids. At room temperature, the lipids presented in SLN are in solid state while the binary lipids in NLC are in solid and liquid state [15, 16]. In particular, SLN is capable of delivering drugs via various parenteral routes to improve biocompatibility and bioavailability of drugs [17–19]. In addition to these advantages, NLC also offers increased drug loading and reduced drug leakage during storage [20, 21]. The NPs combine the safety and stability of liposomes and polymer nanoparticles [22]. The NPs as transdermal delivery carriers are characteristic of simple preparation with low cost, small side effect and easy to administer.

Recently, NPs have become an important means to promote drugs percutaneous absorption by overcoming the barrier effect of SC. However, the mechanism of transdermal permeation is still unclear, and different researchers have their own opinions. Previous studies found that lornoxicam was successfully loaded in SLN and NLC (LRX-SLN and LRX-NLC), and that the penetration rate of LRX-NLC is higher than that of LRX-SLN [23]. N. Iqbal et al. reported that the release rate of olanzapine-SLN was higher than that of olanzapine-NLC [24]. It was also reported that flurbiprofen-NLC was superiority over flurbiprofen-SLN in size particle, drug encapsulation efficiency, stability, in vitro occlusion factor and in vitro percutaneous penetration. In this paper, multi-dimensional researches were carried out to investigate the interactions between lipid nanoparticles and skin. And the visual and dynamic diffusion process of lipid nanoparticles through the skin and skin cells were studied to explore the transport properties of NLC and SLN.

## Results

### Penetration and characterization of TPL-NPs

**Fig. 1:**
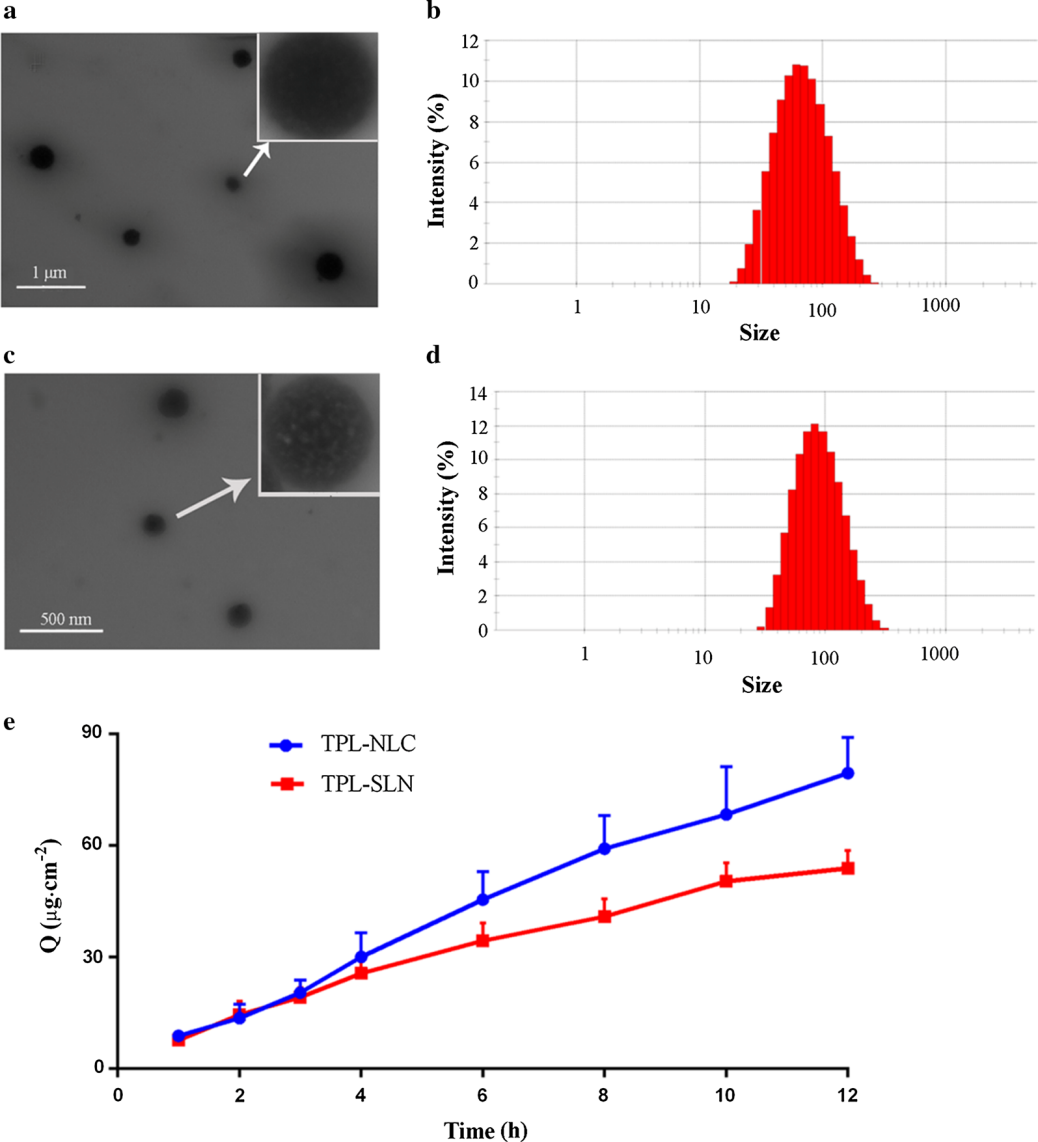
TEM micrographs of TPL-NLC (**a**, ×20,000), DLS analysis of TPL-NLC (**b**, ×50,000), TEM micrographs of TPL-SLN (**c**), DLS analysis of TPL-SLN (**d**). *In vitro* permeation profiles of TPL-NLC and TPL-SLN (**e**). Each value is represented as the mean ± SD (*n *= 5)

The optimal preparation of TPL-NLC was TPL, Compritol 888 ATO, Capryol 90, Tween 80, Transcutol HP, Soya lecithin and redistilled water in the ratio of 1: 7.56: 1.71: 18.54: 6.14: 0.33: 121, while the elements of TPL-SLN were TPL, Compritol 888 ATO, Tween 80, Transcutol HP, Soya lecithin and redistilled water in the ratio of 1: 9.27: 18.54: 6.14: 0.33: 121. The DL% and EE% of TPL-NLC and TPL-SLN was 10.35 ± 1.12%, 9.93 ± 0.98% (P ≤ 0.05), and 97.15 ± 9.46%, 92.81 ± 8.52% (P ≤ 0.05), respectively. The higher DL% of NLC is owned to the fact that ordered lattice of solid lipids (Compritol 888 ATO) is disturbed by the adding liquid lipids (Capryol 90), which help in NLC loading more quality of drug [25]. The morphology and size distribution of TPL-NLC and TPL-SLN is shown in Fig. 1. The morphology of TPL-NLC and TPL-SLN were mostly spherical, uniform (Fig. 1a, c) and the different interior structure might be due to liquid Capryol 90 formed smaller nano accommodations surrounded by solid Compritol 888 ATO in the process of preparing TPL-NLC. As the results of Figure B and Figure D, the size and PDI for TPL-NLC and TPL-SLN were 139.6 ± 2.53 nm, 104.0 ± 1.82 nm and 0.280 ± 0.025, 0.278 ± 0.018, respectively. Besides, Zeta (ζ) potential of TPL-NLC and TPL-SLN was − 36.7 ± 1.39 mV and − 38.8 ± 1.49 mV, respectively.

### In vitro permeation study

The percutaneous permeation profiles of TPL-NLC and TPL-SLN are shown in Fig. 1e. The cumulative amounts of TPL penetrated into receptor medium from TPL-NLC and TPL-SLN at 12 h were 79.51 ± 9.64 and 53.94 ± 5.72 μg cm^−2^, respectively (*P *< 0.05). And the penetration rate of TPL-NLC (6.66 ± 0.92 μg cm^−2^ h^−1^) was higher than that of TPL-SLN (4.23 ± 0.21 μg cm^−2^ h^−1^). As expressed in Table 1, the drug penetrations were determined by linear regression, suggesting that the permeation followed zero order release kinetics [26]. However, TPL was not detected in receiving medium of the control group.

**Table 1:** In vitro penetration fitting curves of the different system (*n *= 5)

Systems	Regression equation	Js (μg cm^−2^ h^−1^)	R^2^
TPL-NLC	Q = 6.66t + 2.40	6.66 ± 0.92	0.9919
TPL-SLN	Q = 4.232t + 6.54	4.232 ± 0.21	0.9824

### Scanning electron microscope (SEM) of skin surface

**Fig. 2:**
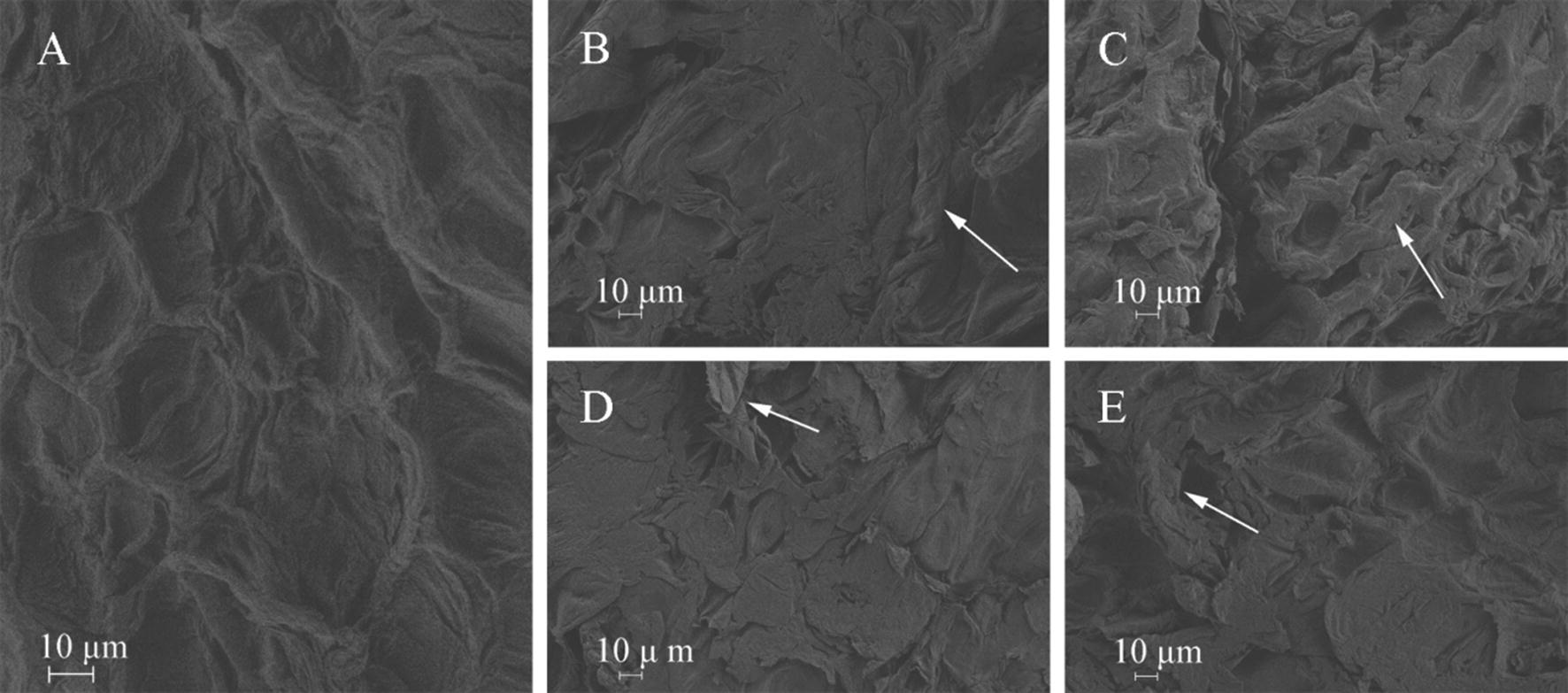
SEM photomicrographs (×500) of skin treated with normal saline (**a**), Blank-NLC (**b**), Blank-SLN (**c**), TPL-NLC (**d**) and TPL-SLN (**e**)

SEM images of skin surface are displayed in Fig. 2. The control skin surface possesses an intact SC with ordered wrinkles (Fig. 2a). Compared with the control group, the skin surface structures of the samples treated with Blank-NLC and Blank-SLN (Fig. 2b, c) was intact with the SC inflated, loose and irregular texture. However, the skin surface of samples treated with TPL-NLC (Fig. 2d) and TPL-SLN (Fig. 2e) was damaged with the SC slices becoming crimped and separating from the skin surface.

### Histopathological (HE) analysis of SC structure

**Fig. 3:**
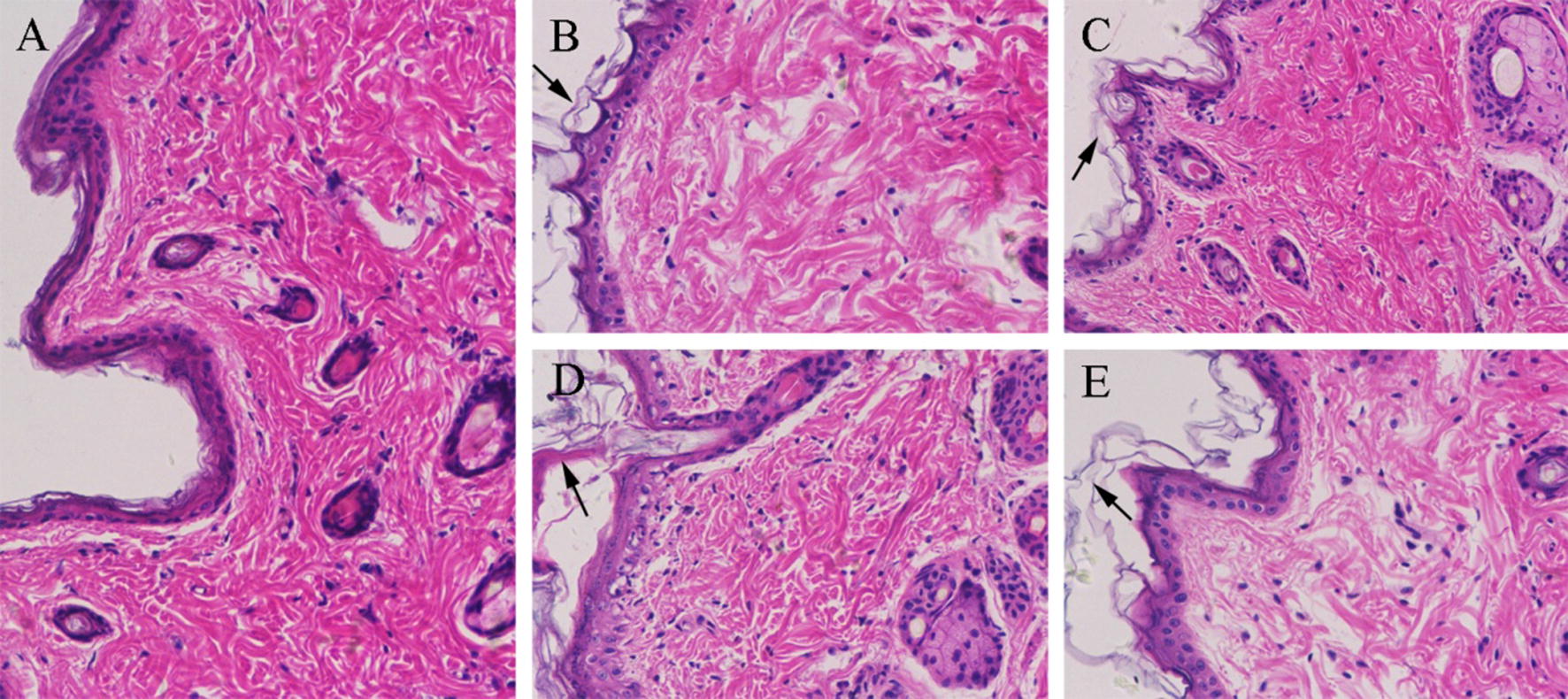
Histopathological photomicrographs (×200) of skin treated with normal saline (**a**), Blank-NLC (**b**), Blank-SLN (**c**), TPL-NLC (**d**), TPL-SLN (**e**)

HE microscopic pictures of skin treated with normal saline and different formulations are shown in Fig. 3. The SC structure of the skin treated with normal saline (Fig. 3a) was compact and the spine cells were interconnected by desmosomes, forming an intercellular bridge, whereas the epidermis of the skin samples treated with Blank-NPs became slightly dilated (Fig. 3b, c). In the groups of TPL-NPs, the SC showed a shedding tendency with a thicker epidermis and larger intercellular spaces. In addition, the spines and basal cells were disordered (Fig. 3d, e). Furthermore, compared with the TPL-SLN group, TPL-NLC has a stronger interaction with skin.

### Differential scanning calorimetry (DSC) analysis of SC thermotropic properties

**Fig. 4:**
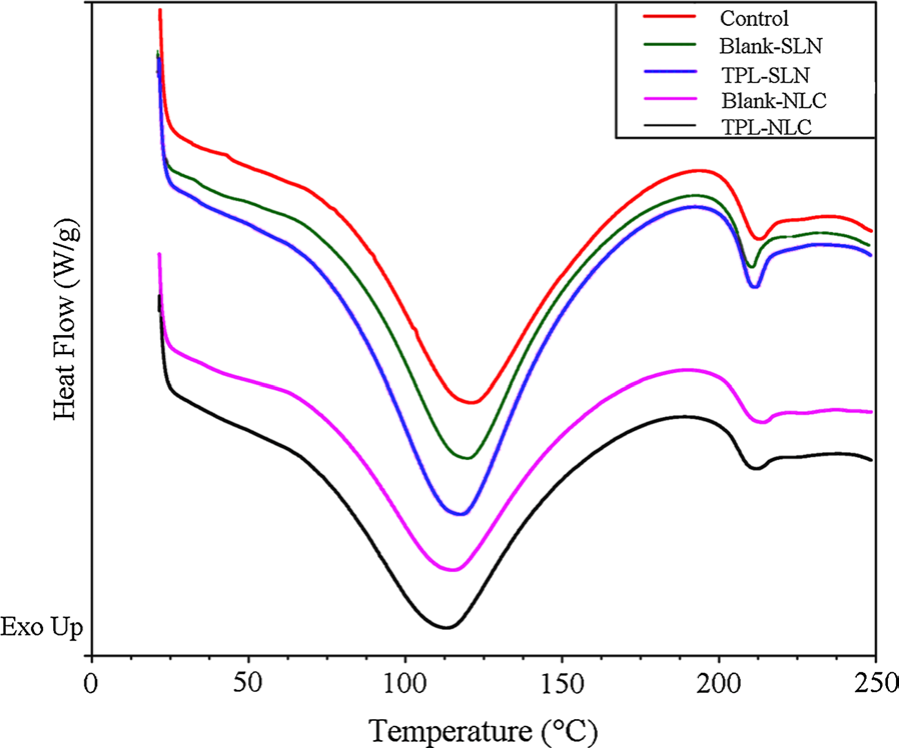
The DSC thermograms of skin tissue treated with normal saline, Blank-NLC, Blank-SLN, TPL-NLC, and TPL-SLN

The DSC curves of the skin samples are shown in Fig. 4 and the characteristic peak in DSC profiles at 110–120 °C was keratin denaturation peak [27]. As shown in Table 2, compared to the control group, the melting point of keratin in the Blank-NPs groups was significantly lower. And the melting point of TPL-NPs groups were further reduced. Besides, keratin melting point of NLC groups was lower than that of SLN groups. Table 2 shows that the enthalpy of different groups has increased to a different degree compared with the control, especially, TPL-NPs > Blank-NPs groups and NLC groups > SLN groups.

**Table 2:** DSC parameters of skin samples treated with different formulations

Samples	Melting temperature (°C)	Heat flow (W g^−1^)	Enthalpy (J g^−1^)
Control	119.65 ± 0.38	− 1.025 ± 0.004	212.9 ± 2.69
Blank-SLN	118.91 ± 0.15	− 1.040 ± 0.070	235.6 ± 1.26
TPL-SLN	116.19 ± 0.32	− 1.182 ± 0.003	289.5 ± 1.06
Blank-NLC	113.69 ± 0.38	− 1.218 ± 0.020	258.5 ± 1.02
TPL-NLC	111.55 ± 0.61	− 1.216 ± 0.019	292.2 ± 2.06

### Fourier transform infrared spectroscopy (FTIR) analysis of SC components

**Fig. 5:**
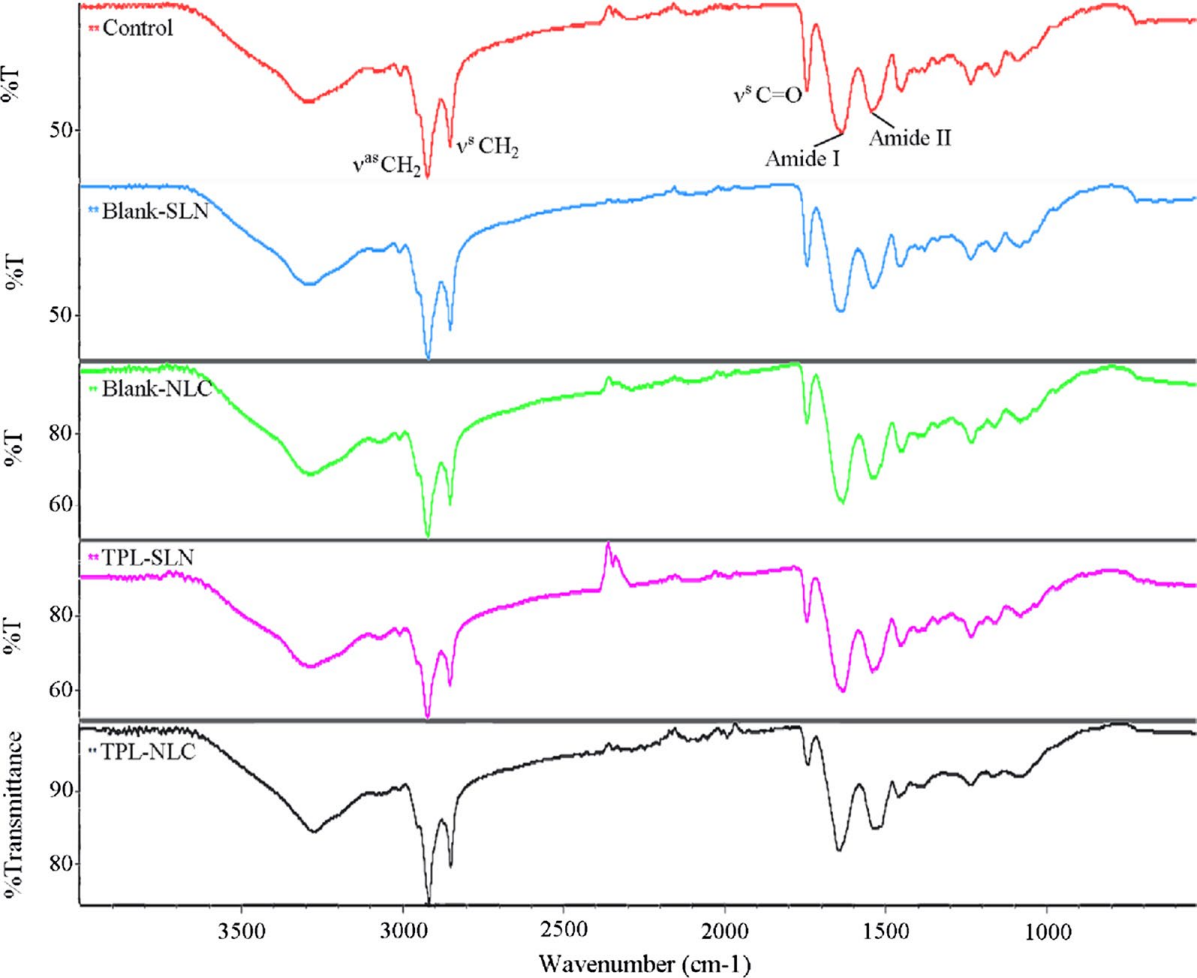
Infrared spectra of skin samples treated with normal saline, Blank-SLN, Blank-NLC, TPL-SLN, and TPL-NLC

The results of the FTIR analysis of the skin samples are presented in Fig. 5 and Table 3. Infrared characteristic absorption peak of skin samples are SC lipids peak (ν^as^ CH_2_, ν^s^ CH_2_, ν^s^ C=O) and keratin peak (Amide I, Amide II) [28–31]. As shown in Table 3, the absorption peak area of the groups treated with Blank-NPs and TPL-NPs was reduced to varying degrees relative to the control. Furthermore, NPs compared to control, TPL-NLC compared to TPL-SLN could reduce the absorption peak area to a greater extent.

**Table 3:** Peak areas of SC lipids and keratin protein of skin samples with different treatment

Samples	Lipids			Keratin protein	
	ν^as^ CH_2_	ν^s^ CH_2_	ν^s^ C=O	Amide I	Amide II
Control	23.011 ± 0.14	12.612 ± 0.054	4.927 ± 0.017	25.496 ± 0.031	19.378 ± 0.192
Blank-SLN	21.929 ± 0.07	12.235 ± 0.032	4.401 ± 0.010	23.809 ± 0.150	17.025 ± 0.090
Blank-NLC	14.009 ± 0.04	7.332 ± 0.057	2.160 ± 0.059	17.602 ± 0.019	13.627 ± 0.367
TPL-SLN	14.181 ± 0.09	7.858 ± 0.016	3.261 ± 0.017	19.927 ± 0.723	15.798 ± 0.234
TPL-NLC	6.138 ± 0.08	3.198 ± 0087	0.875 ± 0.011	7.781 ± 0.075	5.822 ± 0.087

ν^as^ CH_2_, asymmetric C–H stretching; ν^s^ CH_2_, symmetric C–H stretching; ν^s^ C=O, Carbonyl stretching; Amide I, C=O stretching and Amide II, C–N stretching and N–H bending

### Skin distribution of nanoparticles

**Fig. 6:**
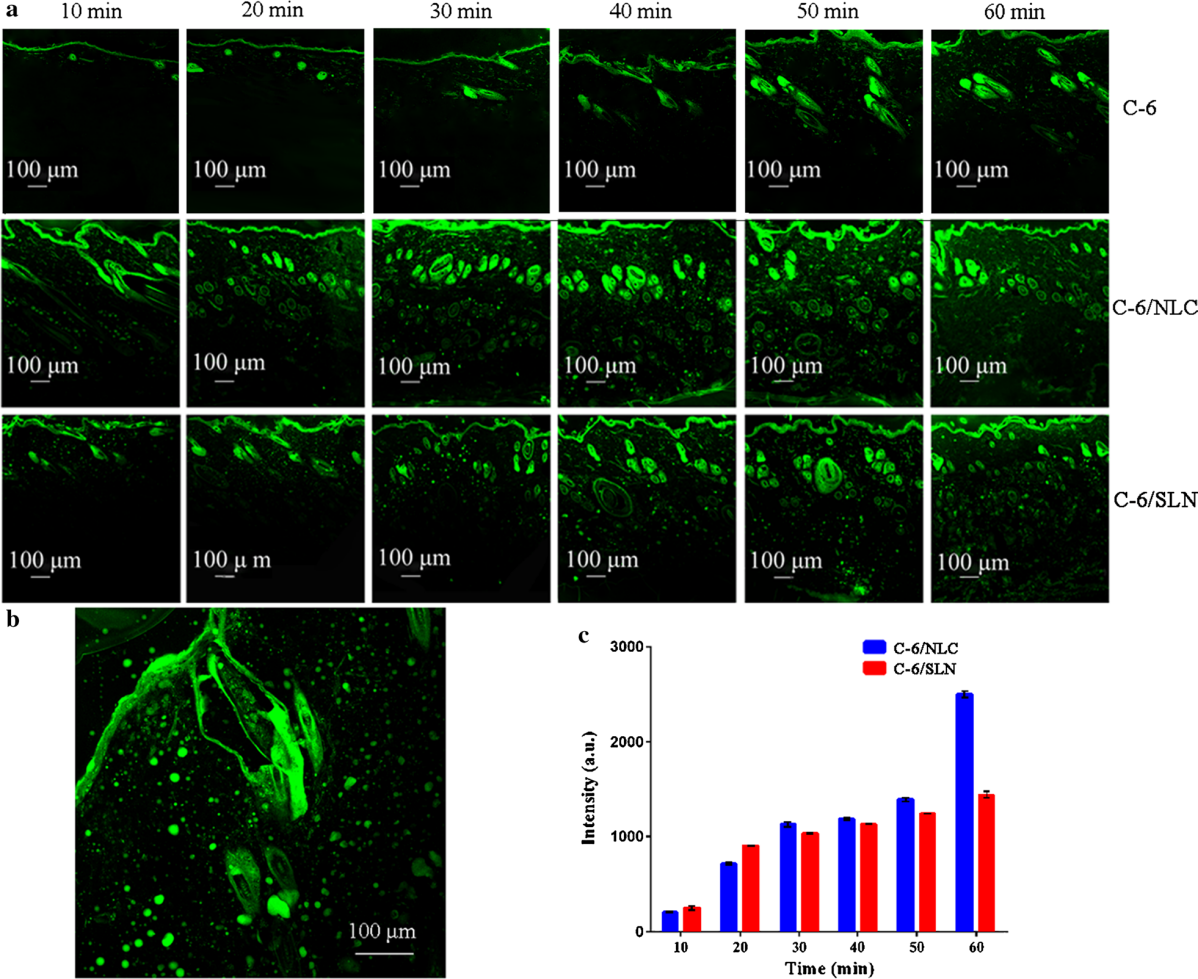
CLSM images (×100) of skin samples treated with free C-6, C-6/NLC and C-6/SLN (**a**), enlarged CLSM Figure (×200) (**b**), fluorescence intensity in receptor fluid at various times (**c**)

The free C-6 and C-6/NPs distribution in skin at different times is shown in Fig. 6a. For the free C-6 group, the fluorescence is only present in the hair follicle after 1 h of administration. For the C-6/NPs groups, the fluorescence signal appeared in the hair follicles initially, and distributed circularly in the entire skin section after treatment with C-6/NPs for 1 h. C-6/NLC permeated into the whole epidermis at 40 min after administration, and the penetration rate was higher than C-6/SLN (Fig. 6a). From the captured intact hair follicle of the skin samples treated with C-6/NLC (Fig. 6b), C-6/NLC could permeate into the whole hair follicles and deposit in the root. Additionally, the fluorescence intensity in the receiving medium (Fig. 6c) was consistent with time-dependent permeation. However, the fluorescence intensity was not detected in the control group.

### Intracellular distribution and cellular uptake of nanoparticles

**Fig. 7:**
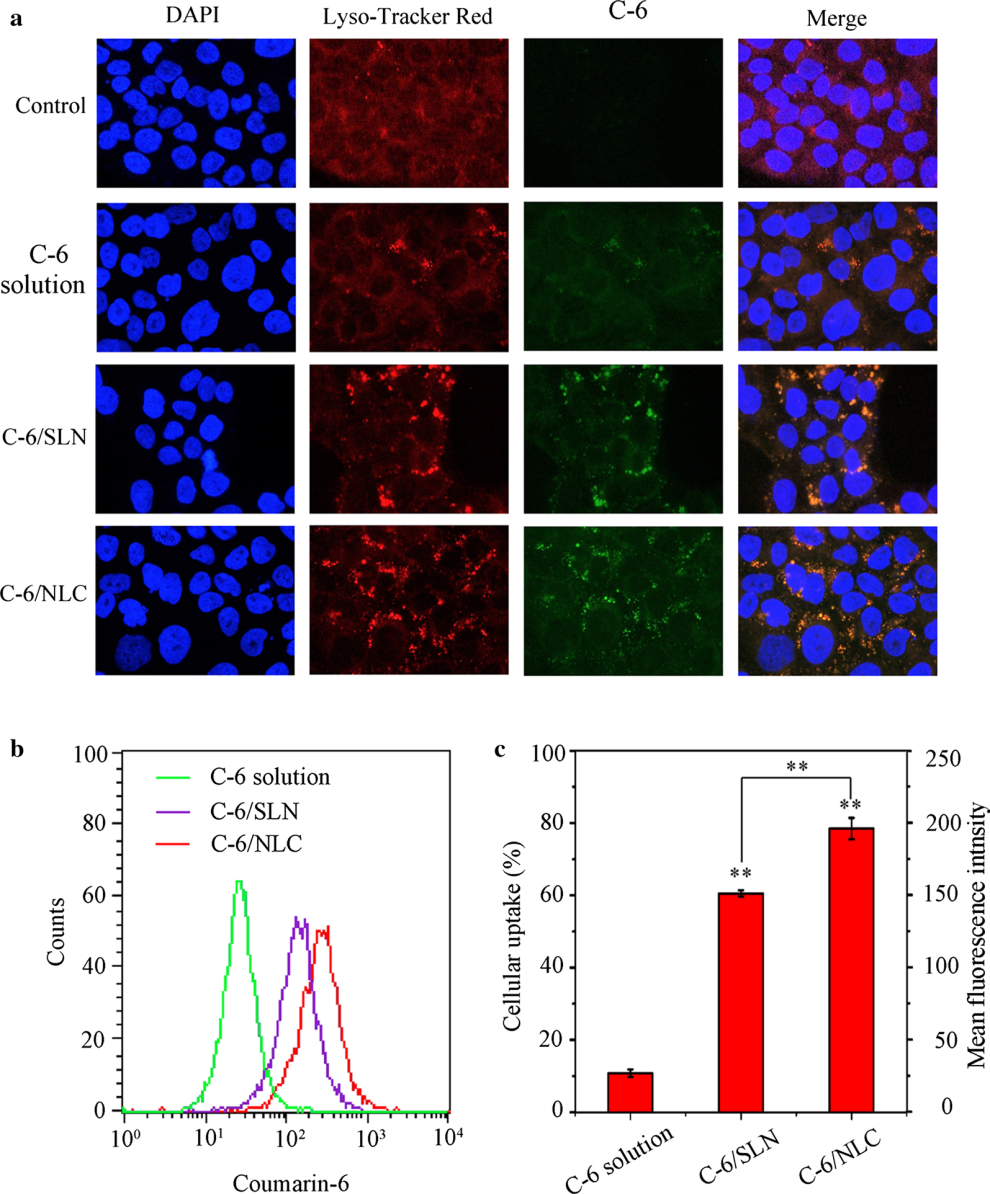
Cellular uptake of C-6 solution, C-6/SLN, C-6/NLC. **a** Intracellular distribution of the formulations in HaCaT cells. **b**, **c**: fluorescence intensity profiles of HaCaT cell treated with C-6 solution, C-6/NLC and C-6/SLN. Each value is represented as the mean ± SD (*n *= 3)

HaCaT cell nuclei (blue), lysosome (red) and C-6/NPs (green) are clearly observed in Fig. 7a. The fluorescence intensity in C-6/NPs groups was higher than C-6 solution group. Meanwhile, lysosome dyed red in control groups was evenly distributed in cytoplasm, while the red light in carrier group was gathered in a corner of endochylema. And the yellow signal was the merger of red (lysosome) and green (C-6). Furthermore, measurable intracellular uptake behaviors of C-6 solution, C-6/NLC and C-6/SLN are shown in Fig. 7b, c. The mean fluorescence intensity of C-6/NLC and C-6/SLN was 7.25 and 5.59-fold higher to C-6 solution. And the fluorescence intensity of C-6/NLC was significantly higher than that of C-6/SLN (P < 0.01).

### Effect of TPL-NPs on the expression of skin inflammatory factors

**Fig. 8:**
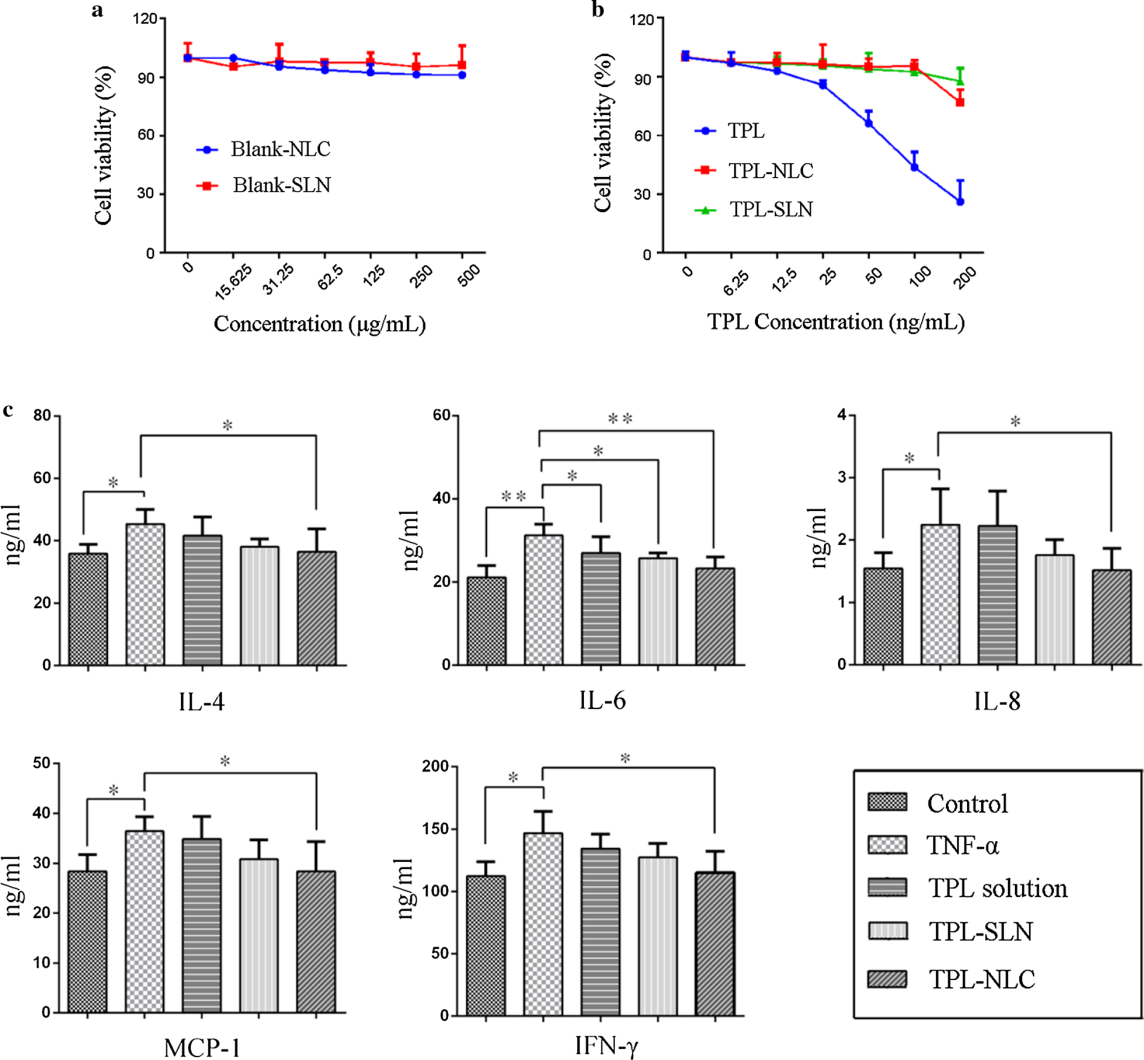
Cell viability affected by Blank-NLC, Blank-SLN (**a**) and TPL solution, TPL-NLC, TPL-SLN (**b**, *n *= 3). Effect of TPL solution and TPL-NPs on the expression of skin inflammatory factors (**c**, *n *= 3). Each value is represented as the mean ± SD, * means *P *< 0.05, ** means *P *< 0.01

As shown in Fig. 8a, empty vehicles showed no cytotoxicity with the cell survival rates higher than 90%, which consist with the reports that most lipid nanoparticles structured with glycerides and non-ionic surfactant are compatible and less toxic to HaCaT cells [32, 33]. What’s more, the viability of HaCaT cells treated with TPL-NLC and TPL-SLN has a negative correlation with its concentration (Fig. 8b). When the cell viability > 90%, the highest concentration of TPL-NPs and TPL solution were 250 μg/mL (the concentration of TPL loaded in TPL-NLC was100 ng/mL) and 12.5 ng/mL, respectively.

As shown in Fig. 8c, HaCaT cells treated with TNF-α over expressed cytokine and chemokine of IL-4, IL-6, IL-8, MCP-1, and IFN-γ compared to the control (P < 0.05). It was found that TPL-NLC could significantly suppress expression skin reaction factors with IL-4 from 45.37 ± 4.06 to 38.9 ± 2.6 ng/mL (*P *< 0.05), IL-6 from 31.23 ± 2.28 to 21.16 ± 2.43 ng/mL (*P *< 0.01), IL-8 from 2.25 ± 0.49 to 1.55 ± 0.22 ng/mL (*P *< 0.05), MCP-1 from 36.67 ± 2.50 to 28.40 ± 2.88 ng/mL (*P *< 0.05), and IFN-γ from 146.81 ± 15.00 to 122.28 ± 10.08 ng/mL (*P *< 0.05), respectively. However, TPL solution and TPL-SLN only inhabited cells expressing IL-6 (*P *< 0.05) and the concentration of IL-4, IL-8,MCP-1, and IFN-γ were not significantly decreased.

### Skin irritation

**Fig. 9:**
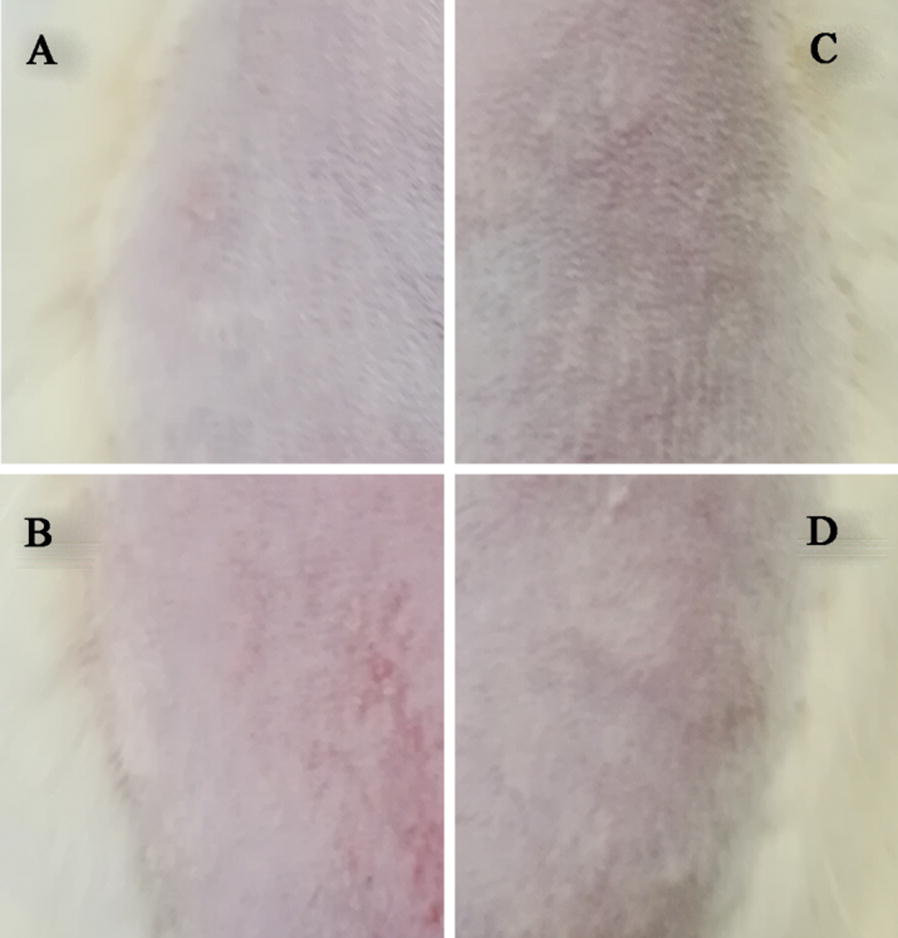
Photograph of the abdomen of Wistar rat exposed to the various formulations: normal saline (**a**), xylene (**b**), TPL-NLC (**c**), TPL-SLN (**d**)

The rat abdominal skin sections treated with normal saline (A), xylene (B), TPL-NLC (C), and TPL-SLN (D) for 12 h are illustrated in Fig. 9. An apparent erythema and swelling was observed in positive groups. Meanwhile, there was no difference in the skin regions treated with TPL-NLC, TPL-SLC and normal saline, which means TPL-NPs develop any skin irritation.

## Discussion

TPL-NLC and TPL-SLN prepared using microemulsion method was evaluated as transdermal drug delivery systems. That the larger size of TPL-NLC might be attributed to the fact that distances between fatty acid chains (Compritol 888 ATO, long-chain fatty acids) could be enlarged by adding different lengths fatty acids (Capryol 90, medium-chain monoglyceride) [34]. Compared to TPL solution, TPL-NPs could permeate into skin effectively and the high permeability rate of TPL-NPs was attributed to the concept that small particles with large surface/volume ratios could effectively interfere with skin [35]. And drug permeation rate and amount from NLC was higher than that from SLN, which may be related to the higher DL% and the added liquid lipid (Capryol 90, which can potentially loosen SC in a manner similar to other surfactants) in TPL-NLC [36].

In this work, skin surface structures were detected by SEM analysis. The SEM photographs displayed that the skin samples treated with TPL-NLC and TPL-SLN were significantly disordered compared to the control group. The results preliminarily indicated TPL-NPs overcame skin barriers by disturbing the ordered structure of the skin surface.

The results of HE studies indicated that the TPL-NPs could permeate into skin by changing SC structure. Both the interactions between mixed surfactants (Tween 80 and Transcutol HP) and skin and lipid exchange between lecithin and SC could increase the skin intercellular space and loosen SC dense structure [37]. Besides, TPL-NPs could inhibit or prevent evaporation, which could improve skin moisture and hydration [38, 39]. Compared with TPL-SLN, TPL-NLC has a stronger interaction with skin, which may be related to the higher DL% and adding liquid lipid (Capryol 90) of TPL-NLC.

DSC was applied to explore the enhanced penetration mechanisms of NPs by analyzing the SC thermodynamic properties. Keratin is a temperature-sensitive substance and the protein conformation was easily changed by external thermal energy, which related to the barrier function of SC. Treated with Blank-NPs, the phase transition temperature of keratin decreased, indicating that the Blank-NPs can effectively penetrate into skin [40]. Compared with the SLN groups, the keratin melting point of the NLC-treated skin is lower, which was due to the melting point of NLC is lower than that of SLN [41]. In addition, the decreased peak height and increased enthalpy of skin treated with NPs formulations directly reflected the changed keratin structure, indicating that lipid nanocarriers could reduce the SC barrier function by changing the helix structure of keratin [42, 43].

FTIR analysis explains the penetration mechanisms of NPs from the perspective of SC structure and components content, because FTIR could provide vibrations information of keratin and SC lipids that are primarily structured SC [44]. The reduced peak areas and peak height of the Amide I and Amide II indicated that the structures of keratin partly transformed to β folding from α helical [45]. Additionally, the peak areas of lipids and keratin were decreased dramatically compared to the control group suggested that TPL-NPs permeability depend on extracting the SC lipid and keratin.

The visual CLSM images (Fig. 6a) demonstrated that it was difficult for free C-6 to penetrate into deep skin. The dynamic transfer process of C-6/NPs in skin was time-dependent and trans-follicular penetration was the preferred pathway because hair follicle openings outward without SC [46]. The hair follicle could serve as drug reservoir for sustained release. The trends of fluorescence intensity were corresponding to the results of dynamic distribution process and the permeation amount of C-6/NLC was greater than that of C-6/SLN, which was consistent with the results of in vitro penetration [47].

Co-localization results of C-6/NPs and HaCaT cells suggested that C-6 loaded in nanoparticles could effectively reduce the efflux of P-glycol protein and could be uptake in the cytoplasm [48, 49]. Most of the nanoparticles enter cells via the endosomal-lysosomal pathway after being phagocytosed. Drugs loaded in nanoparticles were easily degraded and destructed under the acidic and enzyme conditions in endosome/lysosome. The luminance change of Lyso-Tracker Red in the groups of TPL-NLC and TPL-SLN suggested that vesicles were ruptured and the carriers could escape from lysosome. The capacity of rapid endosomal escape of nanoparticles reflected potential anti-inflammatory effect [50–52]. Quantitative study of cell uptake showed that TPL loaded in nanoparticles exhibited better biocompatibility than that of C-6 solution.

The results of Fig. 8a indicated that TPL-NPs are more biocompatible than TPL solutions, because nanoparticles could be internalized by HaCaT cells via phagocytosis or endocytosis while TPL solution was taken up by passive diffusion [53]. The lower DL% of TPL-SLN account for the results that TPL-SLN could only reduce the content of IL-6, while TPL-NLC significantly prevented HaCaT cells secreting inflammatory factors in vitro. The anti-inflammation mechanism of TPL-NPs may attribute to that TPL formulations act on substance P which can inhibit the expression of substance P receptor and reduce the content of skin inflammation factors [54]. These results provided evidence that TPL loaded in NLC significantly inhibit the expression of IL-4, IL-6, IL-8, IFN-γ, and MCP-1 within a biocompatible dose range compared to the TPL solution.

The skin irritation symptoms induced by topical drugs included sweat duct occlusion, itch, erythema and so on. The results of the skin irritation demonstrated that neither the TPL-NLC nor TPL-SLN induced a potential skin irritant effect.

## Conclusions

In summary, TPL-NLC and TPL-SLN constructed in the research possessed spherical, nanoscale morphology and effectively permeate into skin. This study also clarified the enhanced penetration mechanisms and transport properties of nanocarriers, which provide a step for TPL-NPs used in transdermal drug delivery theoretically and practically. The results suggested that TPL-NLC and TPL-SLN are promising agents that could be developed for transdermal transporter.

## Methods

### Materials

Triptolide (TPL) was purchased from Shanghai Yuanye Biological technology co., (Shanghai, China). Compritol 888 ATO (solid lipid), Capryol 90 (liquid lipid), and Transcutol^®^ HP was obtained from Gattefosse (Saint-Priest, France). 4% paraformaldehyde solution was purchased from Wuhan Google biological technology Co., Ltd. (Shanghai, China). Soya lecithin was obtained from Shanghai Taiwei Pharmaceutical Co., Ltd. (Shanghai, China). Coumarin-6 was provided by Sigma-Aldrich (St. Louis, MO). Tween 80 was purchased from Tianjin Guangcheng Chemical Agent Co., Ltd. (Tianjin, China). Enzyme linked immunosorbent assay (ELISA) were obtained from Bender Medsystems GmbH (South San Francisco, USA); Fetal bovine serum (FBS), Dulbecco’s modified Eagle’s medium (DMEM), Lyso-Tracker Red, DAPI, Cell Counting kit-8 (CCK-8), Trypsin were obtained from KeyGEN Bio TECH (Jiangsu, China). The other chemicals and solvents were of analytical reagent grade.

Male SD rats (200 ± 10 g) and Wistar rats (300 ± 20 g) were purchased from the Second Military Medical University. And the animal experiments coincided with the protocols evaluated by the ethics committee of the Second Military Medical University.

### Quantification of TPL

The TPL concentrations were analyzed using RP-HPLC (Agilent 1200 series, USA). The flow rate of the mobile phase composed of methanol and water (48/52, v/v) was 1 mL/min and the detection wavelength was set at 218 nm.

### Preparation of TPL-NLC and TPL-SLN

TPL-NLC was prepared with solid and liquid lipids, surfactant, co-surfactant and water. Solid and liquid lipids were screened based on their ability to dissolve TPL. And the ratio of solid to liquid lipid was determined by measuring the melting distance of the binary in different ratios [41]. The ratio of surfactant to co-surfactant was optimized by structuring pseudo-ternary phase diagram [55, 56]. Additionally, the ratio of TPL to lipids was investigated using EE% and DL% as indicators. Microemulsion technique was used to prepare TPL-NLC and TPL-SLN [57]. Briefly, TPL with optimized lipids, surfactant and co-surfactant were mixed with string at 80 °C for 10 min, and the system was titrated with double-distilled water until the microemulsion system became clarified with opalescence. Then the microemulsion was dispersed into cold water to solidify lipids and structured TPL-NLC. For TPL-SLN, it was prepared with solid lipids, surfactant, co-surfactant and water and the preparation procedures were as of TPL-NLC.

### Drug loading of TPL-NLC and TPL-SLN

Drug loading (DL) and encapsulation efficiency (EE) of TPL-NLC and TPL-SLN were studied using centrifugation technique [58]. Briefly, 1 mL of TPL-NLC or TPL-SLN was added to centrifuge tube and centrifuged at 10,000 rpm at 4 °C for 30 min. Then the supernatant was removed, and the free drug left in the bottom of the centrifuge tube was dissolved in methanol and quantified using HPLC analysis. DL% and EE% of TPL-NPs were calculated by the following equation:

$$ {\text{DL }}\left( \% \right)\, = {{\left( {{\text{W}}_{\text{Total}} - {\text{W}}_{\text{Free}} } \right)} \mathord{\left/ {\vphantom {{\left( {{\text{W}}_{\text{Total}} - {\text{W}}_{\text{Free}} } \right)} {\left( {{\text{W}}_{\text{Lipid}} } \right)}}} \right. \kern-0pt} {\left( {{\text{W}}_{\text{Lipid}} } \right)}} \times \, 100\% $$

$$ {\text{EE }}\left( \% \right)\, = \,{{\left( {{\text{W}}_{\text{Total}} {-}{\text{W}}_{\text{Free}} } \right)} \mathord{\left/ {\vphantom {{\left( {{\text{W}}_{\text{Total}} {-}{\text{W}}_{\text{Free}} } \right)} {\left( {{\text{W}}_{\text{Total}} } \right)}}} \right. \kern-0pt} {\left( {{\text{W}}_{\text{Total}} } \right)}} \times \, 100\% $$

where, the W_Total_ stands the weight of TPL added in NPs. W_Free_ stands the weight of TPL unloaded into NPs and W_Lipid_ stands the weight of the oil phase.

### Particle size, Zeta potential and morphology

Particle size, polydispersity index (PDI) and Zeta potential (ζ) can affect TPL-NPs stability [59, 60]. These parameters of TPL-NPs were measured using a Malvern Zetasizer Nano-ZS (Malvern Instruments Ltd, Worcestershire, England) at room temperature. Prior to measurement, the prepared nanoparticles were diluted 1:20 (sample: redistilled distilled water) to avoid multi-scattering phenomena. And the morphology of the TPL-NPs was visualized by transmission electron microscopy (TEM, 100CXII, Japan).

### In vitro permeation study

SD rats were free feeding for 24 h after abdominal fur removed with depilatory paste. Then the rats were sacrificed and the abdominal skin without subcutaneous fatty tissue was harvested. In vitro permeation study using Franz diffusion cells with an effective diffusion area of 3.14 cm^2^ (Haimen yaohua glass instrument plant, Jiangsu, China) was carried out to evaluate the permeability of TPL solution (control group), TPL-NLC and TPL-SLN. Briefly, the bottom of Franz diffusion cells donor compartment was tightly covered excised abdominal skin and then 0.5 mL of TPL formulations were added into the compartment. The receptor compartment was filled with PBS (containing 20% ethanol, pH = 7.0) to dissolve TPL penetrated from the donor compartment [61]. 1 mL of samples from receiver compartment were quantified using HPLC at the predetermined time intervals (1, 2, 4, 6, 8, 10, 12 h) and the receptor medium was replenished with the same volume, temperature fresh medium.

### Scanning electron microscopy (SEM) of skin surface

SEM (HITACHI S-4800, Japan) was applied to visualize the effects of normal saline (control), nanoparticles without loading TPL (Blank-NPs, including Blank-NLC and Blank-SLN), and TPL-NPs on skin surface microstructure. Excised abdominal skin samples were treated with the different formulations for 12 h using Franz diffusion cells. The treated skin samples were dehydrated using graded ethanol and then deeply dried using lyophilization. After that, the dried skin samples were coated with evaporated gold for conductivity before exposing to SEM.

### Histopathological (HE) analysis of SC structure

The HE study was conducted to analyze the effects of carriers and TPL-NPs on skin SC. The excised skin samples were treated with saline, Blank-NPs and TPL-NPs for 12 h with Franz diffusion cells, followed by washing with normal saline and fixed with 4% paraformaldehyde. And, the samples were embedded into paraffin wax to prepare pathological sections. Then the slices specimens were subjected to hematoxylin/eosin and imaged with light microscope (Leica, Germany).

### Differential scanning calorimetry (DSC) analysis of SC thermotropic properties

DSC (Shimadzu DSC-60, Japan) was used to analyze the SC thermotropic phase behavior. The transdermally treated skin samples were obtained with the method described in the section of SEM analysis. Then the dehydrated skin samples were weighed precisely and added into aluminum crimp cells under nitrogen purge. In DSC studies, testing temperature was set from 20 to 250 °C at a heating rate of 10 °C min^−1^.

### Fourier transform infrared spectroscopy (FTIR) analysis of SC components

FTIR spectrophotometer (Nicolet 6700 FT-IR, Thermo Fisher Scientific, USA) was employed to further investigate the effects of Blank-NPs and TPL-NPs on SC lipids and keratin [62]. The transdermally treated skin samples were obtained with the method described in the section of SEM analysis. Then the samples for infrared analysis were prepared using KBr disc technique and analyzed in the wave number range of 4000–650 cm^−1^ at a resolution of 4 cm^−1^ [63].

### Skin distribution of nanoparticles

Confocal laser scanning microscopy (CLSM) was applied to visualize the nanoparticles dynamic transport processes in the skin. Coumarin-6 (C-6), emitting green fluorescence, was loaded in NPs (C-6/NPs, including C-6/NLC and C-6/SLN) to mimic TPL-NPs [46]. In these studies, excised skin samples were treated with C-6 solution, C-6/NPs for 10, 20, 30, 40, 50 and 60 min using Franz diffusion cells. After administration, the corresponding fluorescence intensity of C-6 permeated in the receptor medium was straightway determined using fluorescence microscopy (Leica, Germany). Simultaneously, the treated skin samples were fixed with 4% paraformaldehyde and exposed to CLSM to investigate C-6 solution and C-6/NPs distribution in skin.

### Intracellular distribution and cellular uptake of nanoparticles

The transport characteristics of NPs labeled with C-6 were further studied at cellular level. HaCaT cells, a human epidermal keratinocyte cell line, were cultured with Dulbecco’s modified eagle’s medium (DMEM) supplemented with 10% fetal bovine serum (FBS) in culture flasks at 37 °C in a humidified atmosphere of 5% CO_2_.

Fluorescence images of cells incubated with different treatments were acquired under a fluorescence microscope (NIKON, Japan) [48]. In these studies, HaCaT cells growing on creep plates were cultured with C-6, C-6/NLC, C-6/SLN for 4 h and cultured with Lyso-Tracker Red for another 30 min to stain the cell lysosome, followed by washing with PBS (pH = 7.4) and fixing with aqueous paraformaldehyde solution (4%, w/v) for 15 min at room temperature. To localize the position of cell nuclei, cells were stained with DAPI before analysis.

The Flow cytometry data were further obtained to quantify the intracellular uptake of C-6, C-6/NLC and C-6/SLN. After incubating with different preparations for 4 h, HaCaT cells were dispersed with trypsin, centrifuged at 1500 rpm for 3 min and resuspended in 200 μL of pre-cooled PBS (pH = 7.4). And the fluorescence intensity of 6000 cells was analyzed by flow cytometry (Becton–Dickinson, San Jose, CA, USA).

### Effect of TPL-NPs on the expression of skin inflammatory factors

Cell viability using a CCK-8 proliferation assay was carried out to select the concentration of different formulations in the studies of anti-inflammatory effect [64]. For the formulations, TPL in DMSO was diluted with complete culture medium into a series of concentrations (6.25–200 ng/mL) while the concentration of TPL-NLC was 15.625–500 µg/mL (the concentration of loaded TPL in TPL-NLC was 6.25–200 ng/mL). The concentration of TPL-SLN, Blank-NLC and Blank-SLN was in line with TPL-NLC. Then the cells in the logarithmic growth phase were cultured with the formulations for 24 h, followed by incubated with fresh DMEM containing 10% CCK-8 for another 20 min at room temperature. A microplate reader (Thermo, IL, USA) was used to determine the cell viability at absorbance of 450 nm.

Tumor necrosis factor alpha (TNF-α) could induce HaCaT cells to secrete inflammatory cytokines and chemokines linked with pathogenesis of cutaneous diseases, including interleukin (IL)-4, IL-6, IL-8, interferon gamma (IFN)-γ and monocyte chemotactic protein (MCP)-1 [54, 65]. To research the anti-inflammatory activity of different TPL formulations, HaCaT cells treated with TNF-α for 4 h were cultivated with DMEM (positive groups), TPL solutions, TPL-SLN and TPL-NLC for another 20 h, respectively. Then ELISA kit was applied to quantify the correlative inflammatory cytokines with the untreated HaCaT cells as negative groups.

### Skin irritation

Skin irritation is the most common dermal reaction for topical delivery. In these studies, Wistar rats treated with different formulations for 12 h were used to research the irritation. The abdomen was divided into four regions after the abdominal fur was removed [55]. The regions treated with normal saline and xylene were as the negative and positive control groups, respectively. And TPL-NLC and TPL-SLN were applied to the regions to examine the skin irritation [66]. After that, the four regions were washed with normal saline and examined erythema signs.

### Data analysis

The results were represented as a mean of at least three experiments with the corresponding standard deviation (SD). Statistical data were analyzed using SPSS software version 17.0 and a statistically significant difference was denoted by the difference probability level (*P* < 0.05). T-test and least-significant difference (LSD) were employed for analysis the statistical data.

## Abbreviations

NPs: nanoparticles
NLC: nanostructured lipid carries
SLN: solid lipid nanoparticles
TPL: triptolide
TEM: transmission electron microscopy
SEM: scanning electron microscopy
HE: histopathological examination
DSC: diferential scanning calorimetry analysis
FTIR: Fourier transform infrared spectroscopy
CLSM: confocal laser scanning microscopy
C-6: coumarion-6
